# Primary solitary hydatid disease of brain in a 16-year-old girl: a case report

**DOI:** 10.11604/pamj.2022.42.195.34744

**Published:** 2022-07-12

**Authors:** Prateek Pulavarty, Paresh Korde, Sagar Rathod, Juhi Patnaik, Rajesh Domakunti, Shivansh Pratap Singh

**Affiliations:** 1 Department of General Surgery, Jawaharlal Nehru Medical College, Wardha, India

**Keywords:** Isolated intracerebral hydatid disease, *Echinococcus*, Dowling's approach, case report

## Abstract

Isolated involvement of brain with hydatid disease is a rare manifestation and occurs in only 1-2% of all Echinococcus granulosus infections. Here we present a young female of 16 years with primary intra cranial hydatid cyst without any extracranial involvement in the liver or lung. The patient was managed surgically and anti-helemthic medications were given, and the patient was discharged. The objective is to report a rare case of primary solitary hydatid cyst of brain. The incidence of isolated hydatidosis of brain is rare and should be considered as a differential diagnosis in endemic areas. Isolated Hydatidosis of brain is managed surgically and has to be removed carefully without spillage with postoperative medication to reduce the risk of recurrence.

## Introduction

Hydatidosis is a zoonotic disease caused by *Echinococcus tapeworm larvae* (metacestodes) infecting humans [1]. *Echinococcus granulosus* is the most prevalent kind of *Echinococcus* infection, while *Echinococcus multilocularis* is the less common variety [2]. Humans are accidental intermediate hosts who become more susceptible to infection after touching tapeworm eggs in soil, dirt, and animal hair [3]. The liver is the most usually infected organ, with a 50% to 77% infection rate, followed by the lungs (9% to 43% infection rate), and involvement of other organs such as the brain, pericardium, muscles, ocular orbit, tibia, kidneys, spleen, and bone marrow is uncommon [4]. Isolated brain hydatidosis is a rare manifestation, occurring in only about 1-2% of all *Echinococcus granulosus* infections. Brain hydatidosis disease is more common in children and young adults [5]. In this article, a rare case of symptomatic isolated brain hydatid in a 16-year-old female child has been reported.

## Patient and observation

**Patient information:** a 16-year-old female who is a resident of Vidarbha region of Maharashtra presented to Acharya Vinobha Rural Hospital, Wardha with complaints of 2 episodes of generalized tonic-clonic seizures with a past history of headache on the left side since last 1 year, she did not give any history of pain in abdomen or shortness of breath.

**Clinical findings and timeline:** general physical and systemic examination of the patient was unremarkable.

**Figure 1:**
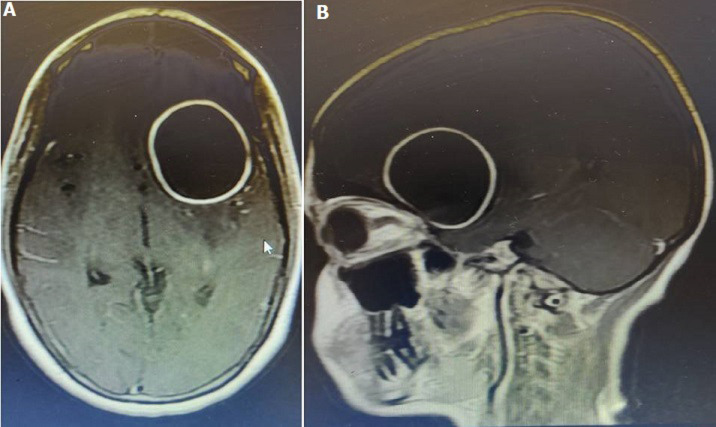
axial view of pre-operative MRI showing well defined circumscribed lesion (A); sagittal view of pre-operative MRI showing well-defined circumscribed lesion (B)

**Figure 2:**
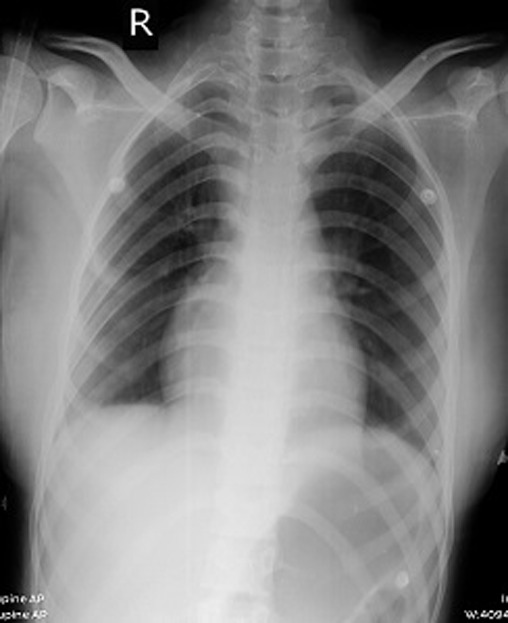
chest X-ray anteroposterior view showing no involvement of lungs

**Diagnostics assessment:** MRI of brain with contrast showed well-defined peripherally enhancing rounded predominantly cystic lesion of size 5 x 4.9 x 4.8 cm in left fronto-temporal region with multiple daughter cysts within a large cyst suggestive of intra-cerebral hydatid cyst. There was also evidence of peri-lesional edema causing mass effect in the form of effacement of left ventricle and midline shift of 9 mm (Figure 1 (A,B)). MRS shows increased lipid lactate in the lesion. Liver enzymes, chest X-ray and USG abdomen showed no evidence of any lung or liver hydatid lesions (Figure 2).

**Figure 3:**
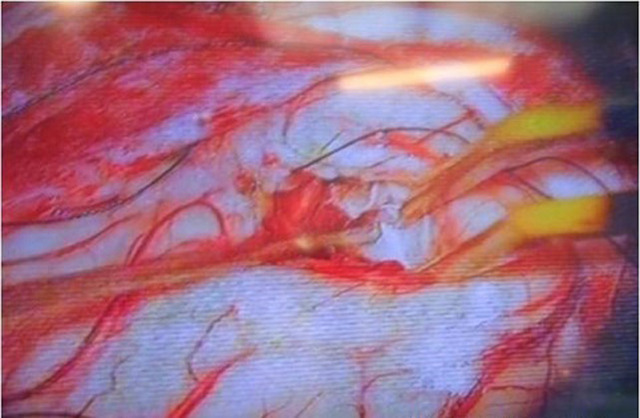
intra operative picture showing capsule of hydatid cyst

**Figure 4:**
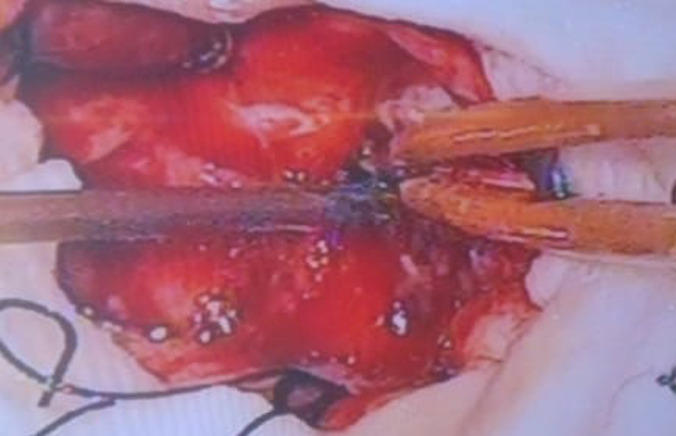
intra operative picture-hydro dissection

**Figure 5:**
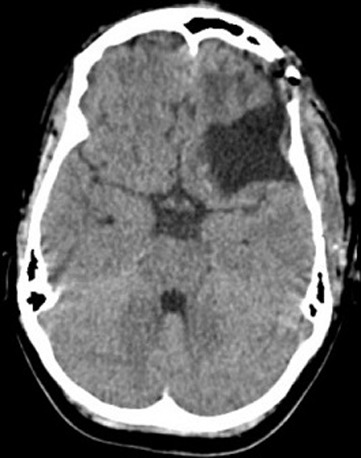
axial view of CT brain showing post-operative changes

**Therapeutic intervention:** fronto-temporal craniotomy was done. A C-shaped incision was given overdue to a matter based over the sphenoid ridge. Cyst was identified (Figure 3) surrounding brain cortex was isolated by covering savlon soaked cotton patties, the cyst wall was separated from the surrounding brain parenchyma by hydro dissection during which the cyst wall was ruptured and contents were spilled into the surrounding tissue, the spilled contents were removed carefully and through was given using hypertonic saline and chemotherapy was applied (Figure 4). The sample was sent for histopathological examination, which confirmed it as a hydatid cyst. Post-operative CT brain with contrast was done, showing post-operative calvarial defect noted in left fronto-temporal bone and no residual cyst (Figure 5). The patient was continued with tab albendazole at a dose of 10 mg/kg twice daily for 14 days for 3 cycles, with the interval between 2 cycles being 1 week post operatively.

**Follow up and outcomes:** follow up on a regular basis of the patient for six months, with regular clinical assessments and radiological monitoring was done, but she was asymptomatic, seizure-free, and showed no evidence of recurrence.

**Patient perspective:** the patient was happy with the successful outcome of the surgery.

**Informed consent:** a written informed consent was obtained from the patient for participation in our study.

## Discussion

Natural transmission of hydatid disease is between humans and animals in which embryonated eggs of *Echinococcus* are infective form for humans. Sexual forms of the parasite are being harbored by canines i.e. definitive host, whereas asexual forms of the parasite are being harbored by wild or domesticated ungulates such as sheep. Humans however act as accidental intermediate host who gets infected from infested definitive host by ingestion of eggs from the food contaminated with their fecal matter [6]. Embryos are released after their enveloping layer is lost when the ingested eggs reach the stomach of humans. These embryos enter into the portal circulation by penetrating through the gut wall and reaches liver where entrapment and encystment of most of the larvae occurs. Few of the penetrated embryos reaches lungs occasionally, and very rarely they overpass capillary filter to enter into systemic circulation and go into sites like brain, heart and bones via hematogenous spread.

One of the infrequent demonstration of hydatids are involvement of Cerebrum constituting only 1-4% among all the hydatid diseases [7]. Based upon the pathogenesis of the disease, these Intracranial hydatid cysts are divided into Primary (single) and Secondary (multiple) [6]. If the presence of cysts in the brain is due to the infestation of the larvae into the brain directly with no evidence of involvement of any other organs in the body, than it is considered to be Primary/Single/Isolated Intracranial Hydatid cyst. Various etiological events causing this type of variant are patent ductus arteriosus and foramen ovale, unique architectonics of brain and defective immunological response, whereas pathogenesis for Secondary/multiple type of variant is thought to be because of hematogenous spread of multiple scolises liberated into circulation after rupture of cardiac hydatid cyst [8] or after surgical/spontaneous/traumatic rupture of solitary cranial hydatid cyst [8]. Secondary (multiple) variant is less frequent compared to Primary (single/isolated) variant. Parietal lobe of supratentorial region is the most common site of manifestation of these hydatid cysts in Brain. Cerebellum, pons, ventricles, cavernous sinus, eyeball and skull are other less frequent sites [9]. Pediatric age group is the most vulnerable for its occurrence. Features of Intracranial Hypertension along with signs of focal neurological deficit is the most common clinical presentation of this disease [10]. In our patient who is a 16-year-old female child, it was found to be Primary (isolated/single) hydatid cyst of Brain as there was no history/investigation revealing the involvement of any other organs like liver, spleen or lung.

The most common diagnostic modalities for cerebral hydatid disease are Computed Tomography (CT) and Magnetic resonance imaging (MRI), while histological testing is still the gold standard. The CT pictures demonstrate a hypodense cystic lesion with neither edema or contrast enhancement perilesionally. T1-weighted pictures show a hypointense cyst, whereas T2-weighted images show a hyperintense cyst with a hypointense halo surrounding its capsule [11]. The daughter cyst has many septations. The presence of a contrast enhancing nodule on CT and MRI specifies the possibility of pilocytic astrocytoma [12] and should be considered in the differential diagnosis. Other differentials that can be considered in a case of space occupying lesion of brain are Arachnoid cysts that are located extra-axially and are not as spherical as other cysts [12] and Brain Abscesses having thick wall and distinctly enhances with contrast, because of the edema around them [12]. Multiple cysts are typical seen in different stages of neurocysticercosis, and they exhibit a “dot in a hole” pattern because the larval scolex may appear as a mural nodule [12].

The aim of surgery for a hydatid cyst is to remove the cyst in toto without rupture, preventing recurrence and anaphylactic reaction. Dowling's approach is currently the most successful and effective method used nowadays. The cyst is delivered using this procedure by lowering the operating table's head and injecting warm saline between the cyst and the surrounding brain parenchyma. To lessen the risk of recurrence after a rupture, surgeons should clean the surgical site with hypertonic saline and apply chemotherapy such as albendazole or mebendazole [13]. Albendazole is administered as a prophylactic chemotherapy adjuvant after surgery, in a daily dose of 10 mg/kg three times a day for four months. Albendazole can also be administered prior to surgery to sterilize the cyst, minimize the risk of allergy, as well as decreasing the cyst wall's tension, lowering the likelihood of leakage during surgery and, as a result, the recurrence rate.

## Conclusion

Primary brain hydatid cysts are uncommon; however, they must be regarded as a differential diagnosis in endemic regions. Surgery is the treatment of choice and must be removed carefully without spillage with postoperative medication to reduce the risk of recurrence.
